# Cell cycle RNA regulons coordinating early lymphocyte development

**DOI:** 10.1002/wrna.1419

**Published:** 2017-02-23

**Authors:** Alison Galloway, Martin Turner

**Affiliations:** ^1^ Centre for Gene Regulation and Expression, School of Life Sciences University of Dundee Dundee UK; ^2^ Laboratory of Lymphocyte Signalling and Development The Babraham Institute Cambridge UK

## Abstract

Lymphocytes undergo dynamic changes in gene expression as they develop from progenitor cells lacking antigen receptors, to mature cells that are prepared to mount immune responses. While transcription factors have established roles in lymphocyte development, they act in concert with post‐transcriptional and post‐translational regulators to determine the proteome. Furthermore, the post‐transcriptional regulation of RNA regulons consisting of mRNAs whose protein products act cooperatively allows RNA binding proteins to exert their effects at multiple points in a pathway. Here, we review recent evidence demonstrating the importance of RNA binding proteins that control the cell cycle in lymphocyte development and discuss the implications for tumorigenesis. *WIREs RNA* 2017, 8:e1419. doi: 10.1002/wrna.1419

For further resources related to this article, please visit the WIREs website.

## INTRODUCTION

Lymphocytes are the cells responsible for adaptive immunity in vertebrates. B cells are the subset of lymphocytes uniquely producing antibodies (secreted immunoglobulins) and recognize antigens through their B cell receptors (BCRs, transmembrane immunoglobulins). In mammals B cells continuously develop from haematopoietic stem cells in the bone marrow throughout adulthood to sustain the mature pool of antigen inexperienced (naïve) B cells. T cells are lymphocytes that recognize antigenic determinants that have been processed and presented by antigen presenting cells through their T cell receptors (TCRs). T cells provide cell‐mediated immunity and help B cells produce antibodies. T cells develop from progenitor cells that have migrated from the bone marrow to the thymus.

Developing B and T cells must execute V(D)J recombination of the DNA encoding immunoglobulin heavy and light chain or of the TCRα and TCRβ loci respectively to produce diverse receptor specificities while avoiding inappropriate DNA damage and maintaining genome integrity. Lymphocytes that produce functional antigen receptors with nonself‐specificities must be positively selected while those producing non‐functional proteins or self‐reactive specificities must be removed. Furthermore, lymphocytes must adapt to a number of distinct niches as they migrate within the bone marrow, blood, spleen, lymph nodes, and other tissues in a developmental stage appropriate manner. To mediate these processes, developing lymphocytes are known to respond to environmental and developmental cues through signal transduction pathways activated by cytokine/chemokine, adhesion receptors and the antigen receptor or its precursor (the pre‐BCR or the pre‐TCR). These regulate gene expression through the expression and activation of developmental stage‐specific transcription factors.^1^ However, it is becoming increasingly apparent that the gene regulatory networks that control lymphocyte development also require the activity of factors that act post‐transcriptionally on RNA. These regulatory networks allow the integration of signaling pathways with the control of mRNA transcription, processing, stability, and localisation.

**Figure 1:**
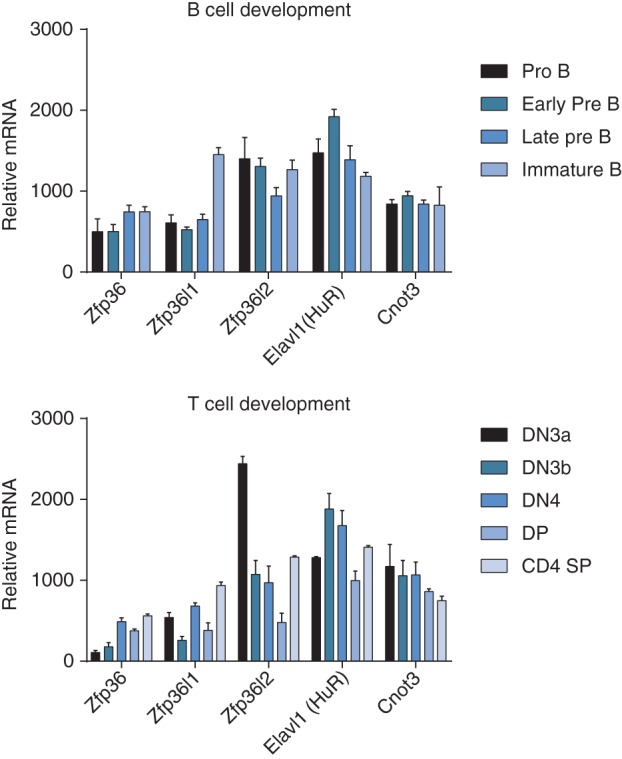
Expression of mRNAs encoding RNA binding proteins in early lymphocyte development. Relative expression of selected mRNAs has been extracted from the immgen database. Source: http://www.immgen.org. Bars represent the mean, and error bars show the standard deviation of three measurements.

Post‐transcriptional control of gene expression is mediated by RNA binding proteins (RBPs) and non‐coding RNAs. Although microRNAs have important roles in lymphocyte development, this review will focus on the role of RBP in early lymphoid development as this topic has received less attention. Regulation through RBP allows signaling events to rapidly influence the fate of existing coding and non‐coding RNAs, thus avoiding the lag time associated with transcriptional changes, and allowing a more diverse and dynamic range of molecular outcomes. Co‐regulated RNAs may comprise sets of transcripts mediating a common function and have been termed RNA regulons.^2^ These can be controlled concurrently by signaling events allowing the cell to coordinate within and between biological processes that might otherwise be considered distinct if they are not coordinately regulated by transcriptional or epigenetic mechanisms. RBP have emerged as a frequent constituent of the proteome and many different protein domains can interact with RNA in a sequence‐specific or ‐nonspecific manner with varying affinities.^3^ The mRNA expression of five RBPs discussed in this review during B and T lymphocyte development is shown in Figure 1, this data was extracted from the immgen immunological genome database.^4^ The RBP‐encoding mRNAs shown: *Zfp36*, *Zfp36l1*, *Zfp36l2*, *Elavl1*, and *Cnot3* are broadly expressed throughout the early stages of lymphocyte development and may exert their effects at many distinct stages.

**Figure 2:**
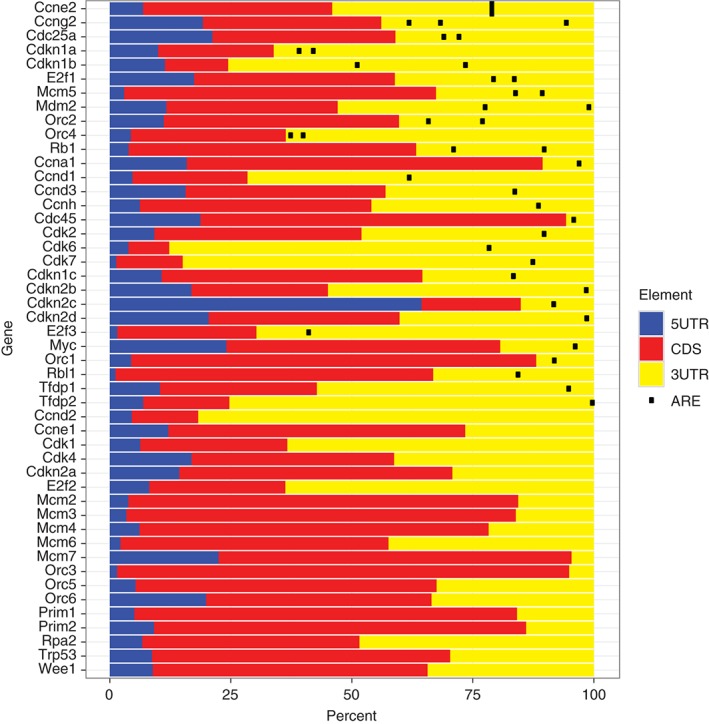
mRNA structure and AU‐rich elements (AREs) within mouse mRNAs encoding factors involved in the G1‐S transition in the cell cycle. The proportion of each transcript that is 5′ UTR (blue), coding DNA sequence (CDS; red) and 3′ UTR (yellow) is shown. AREs in the 3’UTR with the sequence ‘WWAUUUAWW’ where W can be either U or A are marked with black symbols. Transcript information was downloaded from the Ensembl project of genome databases with BioMart software. Where genes were annotated with alternative UTRs or CDS, the longest sequence was selected.

Amongst sequence elements recognized by specific RBPs, the AU‐rich element (ARE), which has the consensus sequence WWAUUUAWW, where W may be U or A, is one of the best studied. AREs are present in as many as 10% of human mRNAs^5^ and interact with a variety of different RNA binding domains. This may allow several RBP to act in concert while decoding cellular signals. Figure 2 demonstrates how AREs are prevalent in the 3′UTRs of mRNAs encoding factors involved in cell cycle progression; note that the UTRs often make up a significant proportion of the transcript suggesting that there could be further regulatory sequences encoded there. Additional regulatory potential may also arise from interactions between the different ARE‐binding proteins, and other transacting factors such as microRNAs. In this manuscript, we will discuss recent progress identifying RBP and RNA regulons that contribute to B and T cell development and consider whether these findings have broader relevance to non‐lymphoid systems and malignancy.

## EARLY B CELL DEVELOPMENT

**Figure 3:**
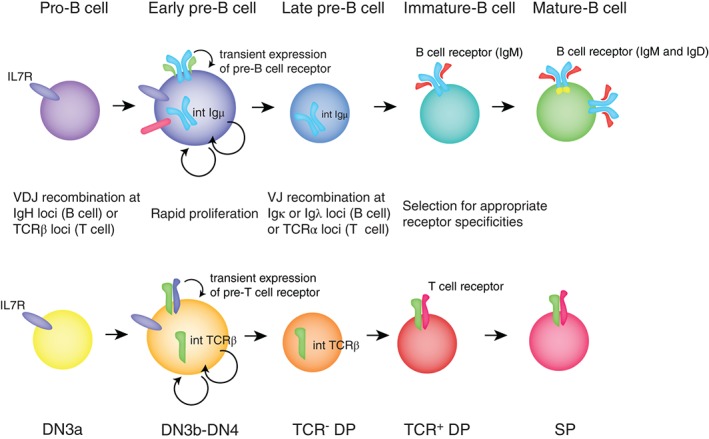
Early stages of B and T lymphocyte development. Important processes during B and T cell development are shown, with the corresponding stages of development. Stages of T cell development are abbreviated to double negative (DN), double positive (DP), and single positive (SP). Briefly, progenitor B and T cells rearrange their IgH or Tcrβ loci respectively, undergo proliferative selection, then rearrange their Igκ/λ or Tcrα loci to form unique antigen receptors.

V(D)J recombination is a process of somatic gene rearrangement unique to lymphocytes where variable (V), diversity (D), and joining (J) gene segments, encoding the complementarity determining regions of the antigen receptors, are recombined by the activity of the recombination activating genes (RAG) to form different coding sequences resulting in a large number of receptor specificities. This allows generation of a diverse repertoire of B and T cells each able to recognize a unique antigen. In B cell development, V(D)J recombination generally occurs first at the immunoglobulin heavy chain (IgH) locus with D to J recombination which is complete at the pre‐pro‐B cell stage of development (Figure 3). Next, V to DJ recombination occurs in a subset of quiescent pro‐B cells. When this generates a functional Igμ protein capable of forming a complex with the surrogate light chains (VpreB and λ5), a cell‐surface signaling receptor, termed the precursor B cell receptor (pre‐BCR), is formed that both promotes a limited, but very vigorous burst of proliferation of the early pre‐B cell, and, through a feedback mechanism, the transition to the late pre‐B cell stage.^6^ Expression of the pre‐BCR induces the transcription factor c‐Myc, which in turn represses p27 (*Cdkn1b*) transcription and enhances the transcription of Cyclin D3, which activates CDK‐4 and ‐6 to drive cell cycle progression in early pre‐B cells.^7^ A later wave of gene expression induced by the pre‐BCR re‐establishes quiescence in late pre‐B cells allowing immunoglobulin light chain recombination at the Igλ or Igκ loci to occur. The transcription factors Aiolos and Ikaros, which repress c‐Myc, are induced and the Dyrk1a kinase limits the expression of Cyclin D3 at this stage.^8^, ^9^ Expression of a light chain leads to the expression of the BCR at the beginning of the immature B cell stage where cells are quiescent and undergo positive and negative selection for appropriate receptor specificities before maturing into naïve B cells. Cells that fail to produce either the heavy or light immunoglobulin chain are eliminated by apoptosis.

The deliberate introduction of DNA strand breaks (DSBs) and the joining of the resected DNA carries the risk that the DNA will not be repaired correctly and it is known that chromosomal translocations and other aberrations do occur in some cells at this stage of development.^10^, ^11^ To minimize this risk, robust mechanisms exist to coordinate V(D)J recombination and cell cycle progression. Signaling through the IL7‐receptor (IL7R) activates STAT5 to repress RAG transcription in proliferating pro‐B cells thus regulating IL7 availability or responsiveness allows the processes to occur in discrete populations.^12^ Furthermore, the RAG2 protein is phosphorylated by cyclin dependent kinase (CDK)‐2 during the G1‐S phase transition leading to its ubiquitination and degradation thus avoiding entry into S phase with the potential to generate RAG‐mediated DSBs.^13^

## THE ROLE OF THE ZFP36 FAMILY IN EARLY B CELL DEVELOPMENT

Recent evidence suggests that the transcriptional and signaling pathways regulating processes that co‐ordinate the dynamic proliferation of developing B and T cells are integrated with post‐transcriptional mechanisms mediated by the ZFP36 family RBP. The ZFP36 family bind to AREs in the 3′UTR of their target mRNAs through a conserved CCCH‐zinc finger structure and recruit deadenylases and decapping enzymes to destabilize their targets.^14^ The ability of these RBP to repress gene expression has been best understood from the study of ZFP36 in the context of cytokine gene expression and inflammation.^15^, ^16^ Less is known about the related ZFP36L1 and ZFP36L2, but studies using conditional mutagenesis in the mouse demonstrate these genes act in a highly redundant manner in developing B and T cells to limit proliferation.^17^, ^18^, ^19^

**Figure 4:**
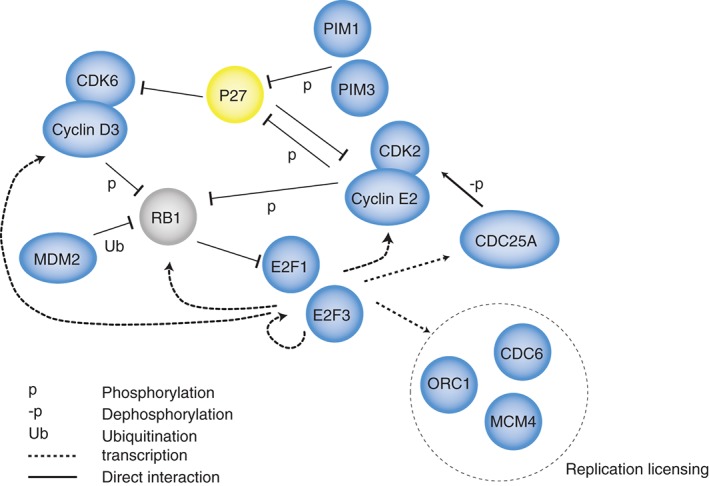
Regulation of the cell cycle by multiple targets of ZFP36L1/L2. ZFP36L1/L2 targets [deduced by AU‐rich element (ARE) sequences in the 3′ UTR or by appearance in ZFP36L1 iCLIP] are shown. Proteins in blue had increased expression in *Zfp36l1 Zfp36l2* KO lymphocytes (either mRNA or protein). P27 in yellow had decreased protein in *Zfp36l1 Zfp36l2* KO lymphocytes. RB1 in gray was unchanged. Interactions between ZFP36L1/L2 targets allow them to cooperate to drive progression into the S phase of the cell cycle.

The mechanistic insight into this process was revealed by the application of individual nucleotide crosslinking and immunoprecipitation (iCLIP) for ZFP36L1. These experiments performed on mitogen activated mature B cells (which bear overlap in their transcriptomes with proliferating pro‐B cells) demonstrated ZFP36L1 bound to AREs in the 3′UTRs of a group of mRNAs encoding proteins involved in the G1‐S transition.^18^ Since the proteins encoded by ZFP36L1 targets act in a cooperative manner to activate the E2F pathway that drives cell cycle progression, and are coordinately regulated by a post‐transcriptional mechanism at the mRNA level they can be considered an RNA regulon (Figure 4). The E2F pathway has a characteristic switching behavior caused by positive feedback; once a threshold of signaling has been reached the G1‐S transition is irreversibly triggered.^20^ The classical E2Fs (E2F1, E2F2, and E2F3) are repressed by retinoblastoma protein (Rb) and this inhibition is relieved when Rb is phosphorylated by the CDKs. Once activated, the classical E2Fs drive transcription of Cyclin E and the phosphatase CDC25A which activates CDK2, thus further increasing Rb phosphorylation.^21^

Overexpression of the RNA regulon repressed by ZFP36L1 and ZFP36L2 amplifies the sensitivity of the E2F pathway thus tipping the balance towards S phase entry and leading to activation of the mechanisms which prevent V(D)J recombination from occurring. The V(D)J recombination defect in *Zfp36l1 Zfp36l2* KO pro‐ and pre‐B cells was rescued by administration of the CDK4/6 inhibitor palbociclib. Conditional overexpression of a GFPZFP36L1 fusion protein inhibited the proliferation of pre‐B cells. Thus *Zfp36l1* and *Zfp36l2* are necessary to establish a quiescent state permissive for V(D)J recombination in pro‐B and pre‐B cells. Furthermore, the increased proliferation of Igμ‐negative pro‐B cells led to defective selection as loss of *Zfp36l1* and *Zfp36l2* allowed some Igμ‐negative cells to express the pre‐B cell marker CD25 indicating that they had bypassed the pre‐BCR checkpoint. However, these cells were apoptotic indicating they had not received the survival signals provided by the pre‐BCR.

The regulation of cell cycle mRNAs by ZFP36 family proteins provides a mechanism by which developing B cells may rapidly change their behavior according to signals from the pre‐BCR and IL7R. Phosphorylation of ZFP36L1 by protein kinase B (PKB) downstream of PI3kinase signaling and through the P38 MAPKAPK2 kinase pathway could repress its ability to promote RNA decay.^22^, ^23^, ^24^ IL7R signaling activates PI3K and the pre‐BCR signals through the P38 pathway which could provide a mechanism through which these surface receptors release the ZFP36L1/2‐mediated inhibition of mRNAs encoding the factors required for S phase entry.^25^ The direct demonstration of these hypothetical links between signaling and RBP in early B cell development will require the development and application of tools and technologies such as phospho‐specific antibodies and proteomics. In addition, *Zfp36* and *Zfp36l1* are transcriptionally repressed by c‐Myc, which is transcribed following IL7R or pre‐BCR signaling providing a further mechanism for regulation of these RBP during early B cell development.^26^ Although kinase and transcription factor targets may differ between different cell types, according to variables such as the subcellular compartmentalisation of proteins and chromatin accessibility, it seems likely that these signaling pathways help to coordinate the cell division cycle with signaling through ZFP36‐family RBP. As the cell cycle is also regulated by other RBP and at the level of mRNA translation, it will be important to establish if additional RBP regulates the cell cycle in developing B cells.^27^

## THYMIC DEVELOPMENT

T cells develop in the thymus in an analogous manner to B cell development (Figure 3). The TCR is formed from two proteins expressed following V(D)J recombination of the TCRα and TCRβ loci. TCRβ is recombined first and forms the pre‐TCR that promotes proliferation and differentiation of T cell progenitors at the β‐selection checkpoint. Conditional KO of *Zfp36l1* and *Zfp36l2* in the T cell lineage leads to the development of T cell acute lymphoblastic leukemia.^19^ Prior to the onset of malignancy *Zfp36l1* and *Zfp36l2* play roles in early T cell development that are very similar to those in B cell development. Thus, in the T cell lineage, *Zfp36l1* and *Zfp36l2* limit proliferation to those cells that have productively rearranged the TCRβ and thus enforce the β‐selection checkpoint.^17^ iCLIP for ZFP36L1 on total mouse thymocytes combined with RNAseq analysis demonstrated that transcripts promoting the G1‐S transition are also a major regulated pathway in these cells. Transcriptomic analysis of the DN3 population (equivalent to pro‐ and early pre‐B cells) demonstrated that the DN3 cells from *Zfp36l1 Zfp36l2* double conditional KOs resembled post β‐selection cells (termed DN3b) more than the quiescent pre‐selection DN3a cells despite not expressing the TCRβ protein. Since DN3b cells are proliferating, the DN3b transcriptional profile includes expression of cell cycle regulators involved in the G1‐S transition, providing further evidence of the control of this RNA regulon by ZFP36L1 and ZFP36L2. NOTCH1 is an important target of ZFP36L1 and ZFP36L2 which is required for driving leukaemogenesis in their absence.^19^ In the β‐selection checkpoint NOTCH1 helps drive proliferation by supporting cellular metabolism by inducing factors including the glucose transporter GLUT1, amino acid transporter CD98 and iron transporter CD71/TFR1.^28^, ^29^ ZFP36 also regulates CD71/TFR1 directly in mouse embryonic fibroblasts, thus the ZFP36 family may help coordinate cell cycle and metabolic regulation around the β‐selection checkpoint.^30^ Notably, as observed in the analogous stages of B cell development, loss of *Zfp36l1* and *Zfp36l2* cannot provide all the signals required for β‐selection. Pre‐TCR signaling increases expression of the IL7R, rendering the cells more responsive to IL‐7,^31^ the *Zfp36l1 Zfp36l2* double conditional KO DN3 cells do not upregulate the IL7R pathway and are more apoptotic than controls.

The increased proliferation of *Zfp36l1 Zfp36l2* DCKO DN3 cells was linked to an increased rate of differentiation consistent with findings that differentiation is linked to the numbers of cell divisions during T cell development.^32^ *Zfp36l1 Zfp36l2* cKO DN3 thymocytes were also found to have increased γH2AX, which marks double strand breaks, and increased phosphorylation of substrates of the ATM/ATR kinases that initiate the DNA damage checkpoint. Despite this, it remains unclear whether the DCKO cells actually have increased DNA damage or whether the DNA damage response is more activated *per se* and it is possible that the phosphatases that regulate γH2AX may be less active in the DCKO cells. If DNA damage itself is increased, this may contribute to the oncogenic transformation of *Zfp36l1 Zfp36l2* cKO thymocytes into T‐ALL.

Unlike the situation in developing B cells, the DNA damage response is able to promote the developmental progression of DN3 thymocytes, and irradiation can do so in the absence of TCRβ , the obligate progression factor.^33^ Moreover, p53 acts as a barrier to the developmental progression of DN3 thymocytes and it too enforces the β‐selection checkpoint.^34^ Some evidence exists to suggest that in thymocytes ZFP36L1 may regulate the DDR and p53 pathways as ZFP36L1 bound to several mRNAs whose protein products had roles in the sensing or resolution of DNA damage, however a direct contribution of these RBP to the DNA damage response of thymocytes has not yet been established. The ubiquitin ligase MDM2 was increased in DN3 cells lacking *Zfp36l1* and *Zfp36l2*. *Mdm2* transcript was increased in these cells, as well as late pre‐B cells from the B cell DCKO and *Mdm2* mRNA was bound by ZFP36L1 in both thymocytes and B cells. Thus, *Mdm2* is likely a direct target of ZFP36l1 and ZFP36L2. MDM2 is a negative regulator of P53 so ZFP36L1 and ZFP36L2 may enhance P53 responses in B and T cell progenitors during the periods in which cell cycle progression is being inhibited and V(D)J recombination is occurring. The DSBs caused by V(D)J recombination induce p38 signaling^35^ which inhibits ZFP36 family RBP, thus we speculate that this mechanism may feedback to decrease p53 activation and prepare the cells for cell cycle progression once the DNA is repaired.

Although there was evidence of increased DNA damage checkpoint signaling in thymocytes lacking *Zfp36l1* and *Zfp36l2*, there was no evidence of a P53 transcriptional response in DN3 thymocytes or late pre‐B cells lacking *Zfp36l1* and *Zfp36l2*. P21 (*Cdkn1a*) mRNA, a well‐known p53 target, was elevated in DN3 thymocytes and late pre‐B cells lacking *Zfp36l1* and *Zfp36l2*, however, this does not imply activation of the p53 pathway in this context as the p21 mRNA is also an ARE‐containing target of ZFP36L1.^18^, ^36^ Given the overlap of the transcripts regulating the cell cycle with those regulating the DDR, it seems probable that ZFP36L1 and ZFP36L2 coordinate the expression of factors involved in cell cycle progression with the expression of factors involved in detecting or resolving DNA damage.

## THE CNOT COMPLEX IN EARLY B CELL DEVELOPMENT

ZFP36 family proteins exert their mRNA‐destabilizing effects in part by recruiting the CCR4‐CNOT deadenylase complex, which interacts with a C‐terminal domain that is conserved among ZFP36 family members through the scaffolding component CNOT1.^37^, ^38^ In addition to CNOT1, this complex contains two deadenylases each with two variants in mammals so the Ccr4‐Cnot complex may contain CNOT6 or CNOT6L and CNOT7 or CNOT8 along with several regulatory subunits. Two studies demonstrate that conditional knockout of the Ccr4‐CNOT complex component *Cnot3* in pro‐B cells (*Cnot3* cKOs) leads to impaired B cell development.^39^, ^40^ Surprisingly, however, the mechanisms described have very little overlap with those affecting B cell development in the absence of *Zfp36l1* and *Zfp36l2*; in the Inoue study CNOT3 was found to destabilize *TP53* mRNA and in *Cnot3* cKOs P53 was overexpressed leading to the induction of apoptosis. P53 mRNA abundance was not affected in pre‐B cells lacking *Zfp36l1* and *Zfp36l2* and there was no induction of P53 transcriptional targets (with the exception of p21 which as noted above is also a ZFP36L1 target) suggesting an alternative RBP is recruiting CNOT3 to the P53 mRNA in these cells. Additionally, no transcript signature relating to cell cycle progression was enriched in *Cnot3* cKO pro‐B cells suggesting that either the CCR4‐NOT complex can destabilize mRNAs independently of the CNOT3 subunit or the ZFP36 family RBPs can interact with other mRNA‐repressive complexes in pro‐B cells. As the Galloway et al. study did not characterize the transcriptome of *Zfp36l1 Zfp36l2* DCKO pro‐B cells comparison between the RNA data in these two studies should be made with the appreciation that each study analyzed a different population of developing B cells.

Knockout of *P53* rescued the apoptotic phenotype of the *Cnot3* cKO pro‐B cells observed by Inoue et al., however, there was still a strong defect in V(D)J recombination and development. This defect was linked to decreased transcription of the IgH V genes and decreased IgH locus contraction in *Cnot3* cKOs. These processes bring together the IgH‐V gene segments and D‐J gene segments making them amenable for recombination.^41^ Inoue et al. demonstrated that the expression of known transcription factors that bring about locus contraction appeared normal in *Cnot3* deficient pro‐B cells. They noted that CCR4‐NOT complex has additional roles in transcription and in nonsense‐mediated decay (NMD) which is required to degrade non‐coding IgH mRNAs which impede further attempts at V(D)J recombination, this could be a mechanism through which CNOT3 might potentiate V(D)J recombination. In contrast, the study by Yang et al. demonstrated reduced expression of PAX5, a transcription factor required for IgH locus contraction, in *Cnot3* cKO pro‐B cells.^42^ Notably they did not observe an induction of P53 in their *Cnot3 RERTcre* cKOs and suggested that the P53 induction observed by Inoue et al. could be dependent on the genotoxic stress induced by Mb1cre interacting with loss of *Cnot3*. Yang et al. demonstrated that CNOT3 interacts with the transcription factor EBF1 and that this interaction is required for the expression of several EBF1 target genes that are essential for B lineage specification and B cell development. A mutant EBF1 that could not interact with CNOT3 bound less efficiently to approximately half of EBF1 target genes. However, neither *Pax5* nor *Ebf1* itself appeared to be sensitive to loss of the EBF1‐CNOT3 interaction. The discrepancy in these results could be due to the difference in developmental stage at which *Cnot3* expression is lost since the transcriptomics was performed on pro‐B cells isolated from RERT2cre‐expressing mice following tamoxifen treatment. CNOT3 could have slightly different roles in the initial induction of *Ebf1* and *Pax5* expression in earlier B cell progenitors. Notably some EBF1 target genes had higher mRNA expression in the absence of the EBF1–CNOT3 interaction, and this was associated with increased transcript stability indicating the role of CNOT3 in gene expression in pro‐B cells is complex and can be repressive or activating depending on the context.

## THE ROLE OF HUR IN EARLY B AND T CELL DEVELOPMENT

The dynamic control of RNA regulons is, in principle, facilitated by the potential for RBP with the same binding specificities to mediate distinct outcomes for the fate of the RNA. The ARE, with its ability to bind multiple different RBP, may be an exemplar of this form of regulation. HuR, encoded by a paralog of the drosophila embryonic lethal abnormal vision gene (*Elavl1*), is a RNA recognition motif (RRM) domain containing, ARE‐binding protein that is proposed to antagonize the destabilizing effects of the ZFP36 family.^43^, ^44^ HuR has been demonstrated to bind to cell cycle mRNAs in an activated T cell line, and the cell cycle is among pathways altered by loss of HuR in activated mature B cells.^45^, ^46^ Additionally, HuR has been demonstrated to promote cell cycle progression in cancer cell lines^47^, ^48^, ^49^ and can interact with a number of transcripts that both positively and negatively regulate cell cycle progression including p27^50^, ^51^, ^52^ and Cyclin D3, the key cyclin for the proliferative expansion of developing lymphocytes.^53^, ^54^

Conditional deletion of *Elavl1* in transplanted bone marrow cells using a tamoxifen regulated Rosa26CreERT2 system resulted in a large reduction in the pro‐ and pre‐B cell compartment and also in immature thymocytes.^55^ By contrast, the deletion of *Elavl1* using Mb1cre, which is active from the pro‐B cell stage onwards, resulted in only a two‐fold reduction in pre‐ and immature‐B cells in the bone marrow.^45^, ^56^ Deletion of *Elavl1* using *Lck‐cre*, which deletes in double negative thymocytes also had a milder effect on the numbers of immature thymocytes.^54^, ^57^ These differences could reflect a critical role for HuR in B cell development prior to the pro‐B cell stage and in the earliest thymocytes. However, it remains unclear to what extent the toxic effects of the combination of tamoxifen and the Rosa26CreERT2 allele^58^ might have contributed to the reductions in pro‐ and pre‐B cells and thymocytes in these experiments.^55^ Interestingly, no cell cycle defect was detected in HuR cKO pro‐ or pre‐B cells suggesting that the cell cycle regulon regulated by ZFP36L1 and ZFP36L2 is independent of HuR in these cells.^56^ Other *Elav* family members do not appear to be expressed in developing lymphocytes thus it may be that other ARE‐binding proteins mediate opposing effects to the ZFP36 family in B cell development or that no such mechanism exists in this context.^4^, ^45^

In human T cells, HuR bound *Ccnd3* mRNA^53^ and promoted expression through an effect on mRNA stability indicating a potential role in proliferation. Furthermore, *Ccnd3* mRNA was found in HuR immuno‐precipitates from mouse thymocytes indicating that HuR may be part of a ribonucleoprotein complex containing *Ccnd3* mRNA.^54^ In a mixture of thymocytes subsets lacking HuR, the abundance of *Ccnd3* mRNA was unchanged but Cyclin D3 protein abundance was reduced. Taken together, these findings are consistent with the suggestion that HuR promotes translation of *Ccnd3* mRNA but the mechanism for this remains unclear. A clear understanding of the role of HuR in the proliferation in early thymocytes is complicated by differences between published studies.^54^, ^57^ Neither study measured directly the proliferation of the TCRβ‐selected DN3 thymocytes by separation of pre‐ and post‐selection DN3 cells. While evidence that HuR may promote P53 expression in thymocytes could indicate a role in limiting differentiation at the DN3 stage. It is also apparent that HuR has role in the selection of thymocytes and in their egress from the thymus.^57^ Moreover, there is evidence that HuR functions in T cell activation and from transcriptome wide studies does so by regulating many pathways.^46^, ^59^, ^60^

## THE ZFP36 FAMILY CELL CYCLE REGULON IN OTHER PHYSIOLOGICAL OR PATHOLOGICAL CONTEXTS

The evidence that ZFP36L1 and ZFP36L2 regulate quiescence in developing B and T lymphocytes is strong. It is also possible that the ZFP36 family RBP regulate quiescence in mature lymphocytes; germinal centre B cells undergo alternating cycles of proliferation and quiescence during affinity maturation and could use these RBP to switch between these two states. T cell activation is accompanied by extensive proliferation that is associated with the acquisition of effector function. Interestingly, a global analysis of 3′UTR shortening in activated T cells suggested a loss of ZFP36 binding sites was associated with T cell activation, consistent with an overall inhibitory function of ZFP36 family proteins in T cell activation.^61^ Additionally ZFP36 family RBP may regulate the cell cycle in more diverse cell types. Deregulation of ARE‐mediated control has been associated with malignant transformation.^62^ Shortening of the 3′UTR by increased usage of cleavage and polyadenylation sites proximal to the translation stop codon, led to loss of AREs from the 3′UTR and increased abundance of the coding transcript. This was associated with increased proliferation in glioblastoma. Interestingly, Cyclin D1, a target of ZFP36 was identified amongst transcripts regulated by this mechanism.^62^ A colon carcinoma cell line lacking *ZFP36L1* overexpressed the targets Cyclin D3 and Cyclin D1 suggesting many target mRNAs are shared across species and different cellular contexts.^18^ The rapid depletion of hematopoietic stem cells in germ‐line knockout *Zfp36l2* mice is consistent with an accelerated stem cell exhaustion phenotype arising from unlimited stem cell proliferation.^63^, ^64^ Loss of quiescence has also been observed in muscle satellite cells deficient in *Zfp36*. In this context, ZFP36 was shown to regulate the transcription factor MyoD which promotes proliferation and differentiation of muscle satellite cells in response to muscle damage, but this ZFP36 target could be part of a broader regulon enforcing quiescence in muscle satellite cells.^65^ ZFP36 may also regulate the balance of quiescence and proliferation in fibroblasts as mouse embryonic fibroblasts lacking ZFP36 proliferate more.^30^ Notably, the targets of ZFP36 in HEK‐293 cells were overexpressed in pre‐B cells lacking *Zfp36l1* and *Zfp36l2* and included many constituents of the cell cycle RNA regulon repressed by these RBP.^18^, ^66^ This suggests that target mRNAs are commonly shared between all three ZFP36 family members, consistent with their highly conserved zinc finger structure. The exact activity of ZFP36 family RBP may still depend on the cellular context as in macrophages ZFP36 has not been determined to regulate the cell cycle.^67^, ^68^ However, during myeloid differentiation ZFP36L1 regulates CDK6, which, in this context, controls differentiation to the monocyte and macrophage lineages.^69^ Thus, the significance of shared targets of these RBP is requires knowledge of the differentiation state of the cell and its transcriptome, as well as the expression of other RBPs in the cell.

The prevalence of AREs within growth factors, cell cycle progression factors, and proto‐oncogenes has prompted the hypothesis that ARE‐binding proteins that limit expression of these transcripts could function as tumor suppressors. There is increasing evidence to support this including results from cell lines,^70^ animal models,^19^, ^26^ and patient studies and this has been reviewed recent by Khabar.^71^ Transgenic expression of ZFP36 inhibited the development of B cell lymphomas in the Eμ‐myc mouse tumor model.^26^ However, a *Zfp36l1* transgene did not reduce disease incidence. This could reflect a difference in the ability of the RBP to act as tumor suppressors or it could reflect a lower magnitude of transgene expression.

**Figure 5:**
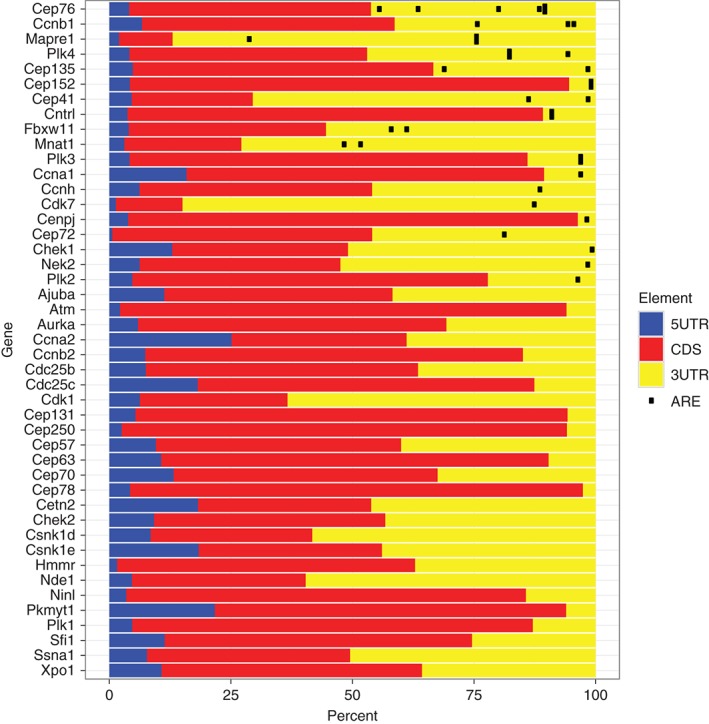
mRNA structure and AU‐rich elements (AREs) within mouse mRNAs encoding factors involved in the G2‐M transition in the cell cycle. The proportion of each transcript that is 5′ UTR (blue), coding DNA sequence (CDS; red) and 3′ UTR (yellow) is shown. AREs in the 3′ UTR with the sequence ‘WWAUUUAWW’ where W can be either U or A are marked with black symbols. Transcript information was downloaded from the Ensembl project of genome databases with BioMart software. Where genes were annotated with alternative UTRs or CDS, the longest sequence was selected.

Loss of *ZFP36* correlated with poor patient outcomes in a number of studies of different cancers^72^, ^73^, ^74^, ^75^ and ZFP36 inhibited cancer cell proliferation.^72^ By contrast, elevated expression of HuR correlated with higher grade breast cancers (recently reviewed by Kotta‐Loizou et al.^76^), and might oppose ZFP36 in this context. Bioinformatics analysis identified a group of mitosis‐related ARE‐containing transcripts that extended into those regulating the G2/M phases of the cell cycle.^73^ Furthermore, the ZFP36 promoted cell cycle arrest is consistent with the notion that transcripts encoding proteins that promote mitosis might also be repressed by the ZFP36 family (Figure 5). Although there was no evidence for an effect on the G2‐M transition in developing lymphocytes lacking *Zfp36l1* and *Zfp36l2*, the level of CDK signaling as the cell exits mitosis can influence the decision to enter G0 or G1 following division^77^ and ZFP36 targets relevant to the G2M transition have also been identified in other systems.^78^ In addition to the cell cycle, additional processes, related to tumor growth, may also be limited by the ZFP36 family including the epithelial‐to‐mesenchymal transition and angiogenesis.^79^, ^80^, ^81^, ^82^, ^83^ Furthermore, the ability of the ZFP36 family to suppress inflammation may contribute to a microenvironment less conducive to tumor development.^84^ It will be interesting to see if in mouse models in which ZFP36 activity is enhanced, either though removal of the inhibitory phosphorylation sites,^85^ or through removal of the auto‐inhibitory feedback that acts via the ARE in the *Zfp36* mRNA 3′UTR,^86^ the development of cancer is forestalled.

Here, we have discussed examples of RNA regulons that coordinate important processes in lymphocyte development. Several challenges must be faced to advance our understanding of RNA regulons in lymphocyte development and other biological contexts. Many RBPs share sequence specificity for simple motifs such as the ARE and this can make studying their functions challenging, for example, all three ZFP36 family members are expressed in developing T and B cells and KO of two family members is required to have dramatic effects on early lymphocyte cell development. This ‘redundancy’ was also observed in lymphomagenesis: T cell acute lymphoblastic leukemia develops in *Zfp36l1 Zfp36l2* double cKOs, but not in the absence of only one family member. In this context, *Zfp36* was unable to compensate for the loss of *Zfp36l1* and *Zfp36l2*. In contrast, although there are four members in the HuR family, only HuR is expressed in B cells, but it may still compete with other RRM‐containing or ARE‐binding RBP from other families. The mis‐expression of the HuR relative HuD in thymocytes expressing constitutively active NOTCH3 caused mis‐splicing of the transcription factor Ikaros (Ikzf1) into short dominant negative forms that promote cell cycle progression and lymphomagenesis,^87^ indicating that related family members may also have very different effects on development. A further challenge is to make sense of the functional interactions between AREs, their binding proteins, and non‐coding RNAs such as microRNAs, long non‐coding RNAs, and circular RNAs. We anticipate that these interactions will either promote or inhibit the expression of protein coding genes and will themselves be highly regulated and relevant to pathology.^88^, ^89^, ^90^, ^91^ If better understood, it is possible that these interactions may be exploited as new therapeutic modalities.^92^, ^93^ Studies describing the roles of RBP tend to focus on one or a few major targets, however, since all RBP investigated so far appear to have many RNA targets it is important to try to understand how effects on several targets help to shape the transcriptome. This is easier when the pathways regulated by the RBP are well described, as is the case with regulation of the G1‐S transition, where many of the interactions between several key proteins are known, aiding our understanding of how they cooperate within an RNA regulon. However, it still remains challenging to carefully validate more than a few key targets. It will also be important to validate the signaling pathways that regulate RBP function in the specific biological contexts where the RBPs are found to function. This is because most of the early work describing signaling and interaction between RBP and effector complexes has been undertaken in transformed cell lines where the RBP may be inappropriately regulated by oncogenes. In this context, it is clear that many signaling pathways converge on RBPs including phosphorylation, protein methylation and acetylation, ubiquitination, prolyl‐isomerization, ADP‐ribosylation, and proteolysis. These may affect the composition and function of ribonucleoprotein complexes in ways that are, with our current state of knowledge, difficult to predict.^94^

There may also be context‐dependent differences in transcript structure caused by alternative splicing and alternative polyadenylation as well as enzymatic modification that allow transcripts to include or exclude certain regulatory elements.^95^ We observed that several annotated transcripts fall short of the full 3′UTR length while investigating the AREs in cell cycle regulators, notably, some of these have since been updated, however, it seems likely that determining transcript structures within the cells of interest will be helpful when considering regulatory sequences. The recognition of the importance of post‐transcriptional regulation of RNA operons should fuel attempts to master these challenges.
